# The relationship between preweaning diarrhea frequency and intestinal permeability and blood glucagon-like peptide-2 concentration in dairy calves

**DOI:** 10.3168/jdsc.2025-0927

**Published:** 2026-03-06

**Authors:** H. Satoh, R. Fukumori, K. Shimada, R. Harada, K. Izumi, K. Chisato, S. Oikawa

**Affiliations:** 1 Department of Veterinary Medicine, School of Veterinary Medicine, Rakuno Gakuen University, Ebetsu, Japan 069-8501; 2 Department of Animal and Bioscience, University of Guelph, Guelph, Ontario, Canada N1G 2W1; 3 The National Federation of Dairy Co-operative Associations (Zenrakuren), Shibuya-ku, Tokyo, Japan 151-0053; 4 College of Bioresource Sciences, Nihon University, Fujisawa, Kanagawa, Japan 252-0880

## Abstract

This study aimed to evaluate the associations of diarrhea frequency on intestinal permeability, plasma glucagon-like peptide-2 (GLP-2) concentration, and growth performance in dairy calves. Twenty-two Holstein calves were monitored from birth to postweaning and classified into high (H) and low (L) diarrhea groups based on the number of days with diarrhea (top and bottom 25%, respectively: n = 6). Fecal consistency was scored on a 4-point scale ranging from 0 to 3, and diarrhea was defined as a fecal score ≥2, with total days of diarrhea ranging from 19 to 36 d in the H group and from 3 to 5 d in the L group throughout the 66-d experimental period. To evaluate the intestinal permeability, the Cr-EDTA test was assessed by oral administration of Cr-EDTA followed by blood sample collections at 28, 42, and 63 d of age. At 0, 1, 5, 28, 42, 49, 56, and 66 d of age, blood GLP-2 concentrations were measured. Although no differences in intestinal permeability were detected between groups, plasma GLP-2 concentrations were consistently higher in the L group throughout the observation period. In addition, despite no differences in solid feed intake between groups, ADG was greater in the L group than in the H group. These findings suggest that plasma GLP-2 concentrations may be associated with postweaning ADG, even in the absence of detectable changes in intestinal permeability.

## Highlights

• Calves with frequent diarrhea showed lower plasma GLP-2 concentration.
• Calves with frequent diarrhea had lower average daily gain despite unchanged dry matter intake.
• No relationship was detected between diarrhea frequency and intestinal permeability.

## Graphical Abstract

**Figure:**
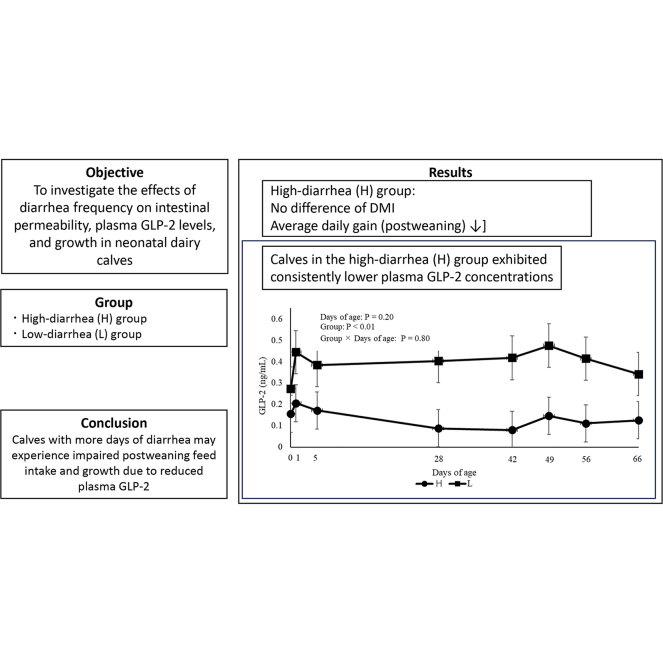
**Summary:**This study evaluated the impact of diarrhea frequency on intestinal permeability, plasma glucagon-like peptide-2 (GLP-2) levels, and growth in neonatal dairy calves. Calves were classified into high- and low-diarrhea groups. Although no differences in intestinal permeability were observed, high-diarrhea group calves showed lower growth rates and GLP-2 levels. These findings suggest that recurrent diarrhea may be linked to reduced GLP-2 secretion, which could impair growth, even without detectable changes in intestinal permeability.

Diarrhea is one of the most prevalent diseases in calves, and it can have long-term effects on their growth and productivity. According to recent national data from the USDA National Animal Health Monitoring System (USDA-APHIS-VS-NAHMS, 2014), digestive disorders, particularly diarrhea, are reported as a major cause of morbidity and mortality in preweaning heifers, highlighting their substantial impact on calf health and farm economics (Heinrichs and Heinrichs, 2011). Several factors may contribute to the occurrence of diarrhea in dairy calves, including developmental changes in gastrointestinal function and structure, as well as an immature immune system in young calves. During the preweaning period, lactose and fat from milk are the primary energy source and are absorbed through the small intestine, similar to nonruminant animals. After weaning, as the gastrointestinal (**GI**) tract develops, solid feed is fermented in the rumen and absorbed as VFA (Khan et al., 2016). This weaning transition process imposes substantial stress on the GI system, making it a critical aspect of calf management (Eckert et al., 2015). Moreover, due to the immature immune system, calves are more susceptible to infections following pathogen exposure. These physiological and immunological challenges increase intestinal permeability, which can result in diarrhea (Araujo et al., 2015). Increased intestinal permeability reflects impaired intestinal barrier function, which plays a critical role in maintaining intestinal integrity and nutrient absorption. In calves, diarrhea impairs intestinal function and development, compromising nutrient absorption and ultimately hindering growth (Soberon and Van Amburgh, 2013; Smith and Berchtold, 2014).

Glucagon-like peptide-2 (**GLP-2**), a gut hormone secreted by L-cells in the distal small intestine, is released in response to stimuli such as VFA, particularly butyrate, and plays important roles in promoting intestinal epithelial cell proliferation, enhancing villus growth, and strengthening intestinal barrier function (Burrin et al., 2003; Inabu et al., 2018). Therefore, GLP-2 may contribute to the regulation of intestinal permeability by maintaining epithelial integrity. Connor et al. (2013) reported that exogenous GLP-2 repairs intestinal damage caused by diarrhea, promoting growth of jejunal epithelium and increasing intestinal weight. In addition, the expression of proglucagon and GLP-2 receptor mRNA has been demonstrated throughout the ruminant gastrointestinal tract and has been shown to be influenced by energy intake, indicating that GLP-2 signaling is nutritionally regulated in ruminants (Taylor-Edwards et al., 2010). Furthermore, Castro et al. (2016) demonstrated that endogenous GLP-2 secretion in milk-fed calves responds to overall nutrient intake rather than glucose provision alone. However, the relationship between GLP-2 secretion, diarrhea frequency, and gastrointestinal function in preweaning calves remains unclear.

Intestinal permeability reflects the passage of molecules through the intestinal epithelium, which is regulated by tight junctions between epithelial cells. It is commonly assessed by measuring plasma or urinary concentrations of Cr-EDTA after administration, which serves as a marker of paracellular transport (Zakia et al., 2025). Glucagon-like peptide-2 has been shown to enhance barrier function by regulating the synthesis of tight junction proteins (Walker et al., 2015), but the relationship between plasma GLP-2 concentrations and intestinal permeability in calves has not been fully elucidated. Therefore, this study aimed to evaluate the relationship between the frequency of diarrhea, intestinal permeability, plasma GLP-2 concentrations, feed intake, and growth performance from birth to postweaning.

This study was conducted at the Field Education and Research Center of Rakuno Gakuen University (Ebetsu, Hokkaido, Japan), and all procedures were approved by the Animal Experiment Committee of Rakuno Gakuen University (approval #VH22C2). A total of 22 Holstein calves (10 males and 12 females) were enrolled. Fecal scores were assessed twice daily using the method of Renaud et al. (2020). Fecal consistency was scored on a 4-point scale (0 = normal; 1 = soft; 2 = runny; 3 = watery), and a fecal score of 2 or greater was defined as diarrhea. Diarrhea was monitored from birth to 66 d of age. Calves in the top 25% for the number of days with diarrhea were classified as the high-diarrhea (**H**) group (n = 6; 4 males and 2 females), whereas those in the bottom 25% were classified as the low-diarrhea (**L**) group (n = 6; 1 male and 5 females). The remaining 10 calves with intermediate diarrhea frequency were excluded from statistical comparison. Birth BW did not differ between groups, averaging 42.9 ± 2.56 kg in the H group and 43.0 ± 3.24 kg in the L group. Similarly, serum IgG concentrations after 24 h of birth were comparable between groups (25.6 ± 5.43 g/L in the H group and 24.8 ± 6.86 g/L in the L group).

All calves received 500 g of commercial colostrum replacer (Good Start Premium, assured value of 264 g IgG/kg on powder basis; Zenrakuren, Tokyo, Japan) dissolved in 2 L of warm water (43°C) and fed via an esophageal tube within 6 h after birth, followed by a second feeding 8 h later. Calves were individually housed in pens (2.1 m × 1.0 m; 2.1 m^2^/calf) separated by steel farm gates. From d 1 to d 5, calves were fed transition milk replacer. The same calves were used as in a currently submitted manuscript by K. Shimada, R. Harada, H. Satoh, R. Fukumori, M. Steele [The National Federation of Dairy Co-operative Associations–Zenrakuren, Tokyo, Japan], and K. Izumi, and were raised under identical management and feeding conditions. The transition milk replacer was divided into 3 groups (milk replacers with high or medium globulin, or without globulin) as described in the submitted manuscript by K. Shimada, R. Harada, H. Satoh, R. Fukumori, M. Steele [The National Federation of Dairy Co-operative Associations–Zenrakuren, Tokyo, Japan], and K. Izumi. Feeding transition milk replacers with different globulin concentrations did not result in significant differences in serum IgG concentration, GLP-2, BW, or fecal score. In the H group, 2 calves received the high-globulin replacer, 2 received the medium-globulin replacer, and 2 received the low-globulin replacer. In the L group, 4 calves received the high-globulin replacer and 2 received the medium-globulin replacer. From d 6 of age, they were fed milk replacer (Calftop EX Black; Zenrakuren; CP 29.7% and crude fat 22.6% on a DM basis) diluted 1:5 with warm water (16.7% wt/vol), twice daily at 0700 and 1600 h using a bucket.

Milk replacer, on a powder basis, was fed at 900 g/d until d 14, 1,200 g/d from d 15 to 42, and then tapered to 800 g/d from d 43 to 49, and 600 g/d from d 50 to 56. Calves were completely weaned at d 57. Calves had ad libitum access to water and calf starter from d 1. Calf starter intake was limited to a maximum of 3.0 kg (as-fed basis) per day, and hay was offered ad libitum. Body weight was measured weekly, and feed intake was calculated daily by measuring orts before the morning feeding.

Blood samples were collected before feeding on d 0, 1, 5, 28, 35, 42, 49, 56, and 66 of age. On d 28, 42, and 66, Cr-EDTA was administered to evaluate intestinal permeability. The prepared Cr-EDTA solution was given at a dose of 5 mL/kg BW (178 mg/kg as Cr-EDTA). During the milk feeding period, Cr-EDTA was mixed with milk replacer, and after weaning, it was administered using a feeding bucket. Blood samples were collected from the jugular vein via an indwelling catheter immediately before Cr-EDTA administration and 4 h after administration, as changes in plasma Cr-EDTA concentrations can be used to estimate intestinal permeability (Zakia et al., 2025).

Immediately after collection, blood was transferred into sodium heparinized vacuum tubes (Terumo, Tokyo, Japan) and stored at 4°C. Within 1 h, samples were centrifuged at 1,940 × *g* for 15 min at 4°C. The resulting plasma was aliquoted into microtubes and stored at −30°C until analysis.

Plasma GLP-2 concentrations were measured by a time-resolved fluorescence immunoassay using a rabbit anti-aggregated GLP-2 antibody, as described by Elsabagh et al. (2017). The intra- and interassay CV were 2.9% and 8.7%, respectively. Determination of serum Cr concentration was conducted based on the method of Ezaki et al. (1991). One gram of serum was weighed into a digestion flask, water and concentrated nitric acid were added, and the mixture was heated until the liquid volume was reduced to one-third of the original volume. The flask was removed from the heater, concentrated nitric acid and hydrogen peroxide solution were added again, the mixture was heated and then removed from the heater, and 1 mL of hydrogen peroxide solution was added. The mixture was heated, and hydrogen peroxide solution was added. This operation was repeated until the sample became transparent. The sample was made up to 100 mL with water and subjected to measurement. Cr concentration was measured using the furnace of an atomic absorption spectrophotometer (Analyst 800, PerkinElmer, Kanagawa, Japan). The first step of the process was conducted at 110°C, with a lamp time of 1 s, a dwell time of 30 s, and a gas flow rate of 250 mL/min. The second step was conducted at 130°C, with a lamp time of 15 s, a dwell time of 30 s, and a gas flow rate of 250 mL/min. This was followed by ashing at 1,500°C (lamp time 10 s, dwell time 20 s, gas flow rate 250 mL/min). Atomization was performed at 2,300°C under the following conditions: 0 s lamp time, 5 s dwell time, and 0 mL/min gas flow rate. Finally, a cleanout was performed at 2,450°C under the following conditions: 1 s lamp time, 3 s dwell time, and 250 mL/min gas flow rate. The recovery rate with the addition of the Cr standard was 103%. The preparation of Cr-EDTA followed the method of Bertens et al. (2024), and the Cr analysis was conducted by atomic absorption spectrophotometry. Intestinal permeability was assessed by calculating the difference in plasma Cr-EDTA concentrations before and after administration.

All data are expressed as LSM ± SEM. The sample size calculation was based on an assumed CV of 10% for plasma GLP-2 concentrations, derived from previous literature investigating gut hormones in calves. With an α of 0.05 and a power of 0.80, a sample size of 6 per group was determined to be sufficient to detect a minimum 15% difference between the groups. Data were analyzed by repeated measures using mixed models in JMP (version 13.2.1, SAS Institute Inc., Cary, NC). For plasma GLP-2 concentration, serum Cr-EDTA concentration, DMI, ADG, and number of days with diarrhea, the model included age, group, sex, and the group × age interaction as fixed effects, with calf included as a random effect. Multiple comparisons among treatment groups were performed using Tukey's test. Statistical significance was declared at *P* < 0.05, and a tendency was noted at 0.05 ≤ *P* < 0.10.

Figure 1 shows changes in plasma GLP-2 concentrations in calves classified by the number of days with diarrhea (fecal score ≥2). Plasma GLP-2 concentrations were significantly higher in the L group than in the H group (group effect: *P* < 0.01), whereas no significant group × age interaction was detected. Figure 2 presents the difference in blood Cr-EDTA concentrations before and after administration. No significant effects of group, age, or their interaction were observed for the difference in blood Cr-EDTA concentration, indicating no detectable difference in intestinal permeability between groups. Table 1 summarizes starter DMI, hay DMI, ADG, and the number of days with diarrhea (fecal score ≥2) by group. No significant group effect was observed for starter or hay DMI. Average daily gain was significantly greater in the L group than in the H group (group effect: *P* = 0.04). The number of diarrhea days was significantly higher in the H group than in the L group (group effect: *P* < 0.01). Sex had no significant effect on any of the measured variables.

**Figure 1:**
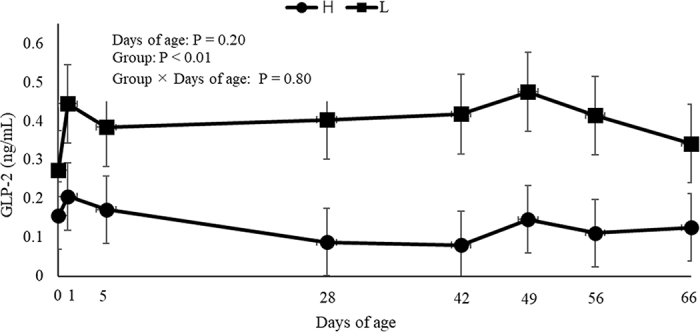
Least squares means ± SEM of plasma GLP-2 concentrations in calves classified by diarrhea status (number of days showing fecal score ≥2). Calves were classified based on the number of days with diarrhea, defined as a fecal score of 2 or greater (fecal score: 0 = normal, 1 = soft, 2 = runny, and 3 = watery), during the experimental period. The top 25% were assigned to the high-diarrhea group (H; n = 6), and the bottom 25% to the low-diarrhea group (L; n = 6).

**Figure 2:**
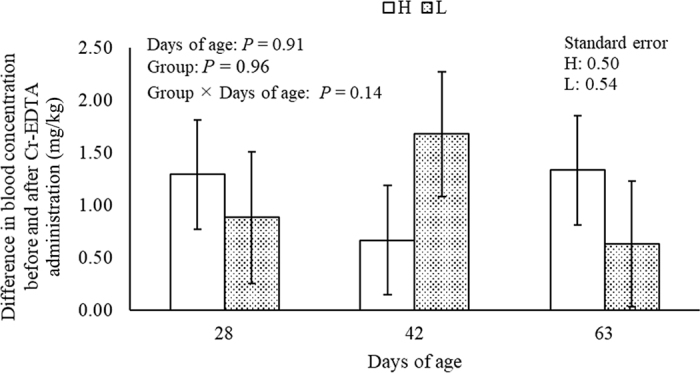
Least squares means ± SEM of the difference in blood Cr-EDTA concentration (mg/kg) between pre- and postadministration in calves classified by diarrhea status (number of days showing fecal score ≥2). Calves were classified based on the number of days with diarrhea, defined as a fecal score of 2 or greater (fecal score: 0 = normal, 1 = soft, 2 = runny, and 3 = watery), during the experimental period. The top 25% were assigned to the high-diarrhea group (H; n = 6), and the bottom 25% to the low-diarrhea group (L; n = 6).

**Table 1:** Comparison of DMI and ADG by diarrhea frequency in dairy calves (LSM ± SEM)

Item	Days of age	Group^1^		*P*-value^2^		
		H	L	Group	Days of age	Group × days of age
Starter DMI (kg/d)	1–5	−0.01 ± 0.10	−0.01 ± 0.12			
	6–14	0.00 ± 0.10	0.01 ± 0.12			
	15–28	0.08 ± 0.10	0.10 ± 0.12			
	29–42	0.26 ± 0.10	0.26 ± 0.12			
	43–49	0.65 ± 0.10	0.78 ± 0.12			
	50–56	1.19 ± 0.10	1.32 ± 0.12			
	57–66	2.10 ± 0.10	2.41 ± 0.12	0.48	<0.01	0.40
Hay DMI (kg/d)	1–5	−0.01 ± 0.05	0.03 ± 0.05			
	6–14	−0.01 ± 0.05	0.03 ± 0.05			
	15–28	−0.01 ± 0.05	0.04 ± 0.05			
	29–42	0.02 ± 0.05	0.07 ± 0.05			
	43–49	0.09 ± 0.05	0.13 ± 0.05			
	50–56	0.12 ± 0.05	0.19 ± 0.05			
	57–66	0.21 ± 0.05	0.34 ± 0.05	0.24	<0.01	0.94
ADG (kg/d)	0–14	0.42 ± 0.11	0.52 ± 0.11			
	15–27	1.00 ± 0.10	1.01 ± 0.11			
	28–41	1.09 ± 0.10	1.14 ± 0.12			
	42–55	0.91 ± 0.11	1.16 ± 0.11			
	56–65	0.87 ± 0.11	1.36 ± 0.11	0.04	<0.01	0.23
Days with diarrhea^3^	0–14	5.51 ± 0.80	0.98 ± 0.82			
	15–27	5.18 ± 0.80	−0.35 ± 0.82			
	28–41	4.68 ± 0.80	−0.19 ± 0.82			
	42–55	3.68 ± 0.80	0.81 ± 0.82			
	56–65	4.68 ± 0.80	1.15 ± 0.82	<0.01	0.66	0.49

1 Groups: high diarrhea (H) = number of days with a fecal score of 2 or higher, top 25% (n = 6); low diarrhea (L) = number of days with a fecal score of 2 or higher, bottom 25% (n = 6).

2 *P*-values represent the results of mixed-effects model analyses assessing the effects of group (H, L), days of age, and their interaction on each measured variable across the entire experimental period.

3 Days with diarrhea: days with a fecal score of 2 or greater.

Intestinal permeability was assessed by the increase in plasma Cr-EDTA concentrations following administration. It is regulated by tight junctions between intestinal epithelial cells and serves as an indicator of how permeable the intestinal mucosa is to various substances (Zakia et al., 2025). When this barrier function is compromised by inflammation or exposure to pathogens, toxins and microbes can more easily enter the body, potentially triggering systemic inflammation and diarrhea (Araujo et al., 2015). Chronic diarrhea has also been shown to impair nutrient absorption, resulting in growth retardation and reduced future productivity, such as decreased milk yield (Soberon and Van Amburgh, 2013; Smith and Berchtold, 2014). Therefore, maintaining intestinal permeability is essential for both growth and disease prevention. In the present study, no significant differences in intestinal permeability were observed between groups or across different ages. This lack of detectable change may be primarily attributed to methodological limitations compared with previous studies. Specifically, we measured plasma Cr-EDTA concentrations at a single time point (4 h after administration), which may not have captured transient changes or cumulative differences in intestinal barrier function. In contrast, other studies that detected increased permeability often utilized cumulative urine collection over 6 to 24 h to integrate total marker passage (Wilms et al., 2024). Pisoni et al. (2021) reported that a single blood sample collected 4 h after administration may be sufficient for evaluating intestinal permeability tests; however, they detected differences between calves under satiated or 9-h fasting conditions, which differed from the conditions in this study. Therefore, although the consistent Cr-EDTA levels might suggest limited physiological stress during the weaning transition, it is more likely that our sampling protocol lacked the sensitivity required to detect subtle alterations in gut permeability in these calves.

Glucagon-like peptide-2 promotes gastrointestinal tract development and enhances intestinal barrier function in calves. Additionally, studies in mice and rats have shown that GLP-2 suppresses increased intestinal permeability and inflammation in the gastrointestinal tract, highlighting its importance in the prevention of diarrhea and support of gut function (Maruta et al., 2020; Deng et al., 2021). Plasma GLP-2 concentrations were consistently higher in the L group throughout the observation period. However, the present study cannot determine whether frequent diarrhea suppressed GLP-2 secretion or whether calves with inherently higher GLP-2 concentrations were less susceptible to diarrhea. Notably, plasma GLP-2 concentrations were numerically higher in the L group already at birth (d 0), suggesting that differences in GLP-2 concentrations may reflect baseline differences in intestinal health rather than a direct preventive effect on diarrhea. This study is observational in nature and therefore cannot establish causation. To clarify these relationships, further verification, including interventional studies, is necessary.

In the present study, no significant differences in DMI were observed between the H and L groups throughout the experimental period. Nevertheless, ADG was significantly (*P* = 0.04) greater in the L group than in the H group. These findings indicate that the observed difference in growth performance was not attributable to differences in feed intake. Calves experiencing more frequent diarrhea may exhibit reduced growth efficiency despite similar nutrient intake, potentially due to impaired nutrient absorption, increased intestinal inflammation, or increased metabolic costs associated with immune activation. In this context, the lower plasma GLP-2 concentrations observed in the H group may reflect compromised intestinal function rather than differences in feed intake. Although GLP-2 has been implicated in supporting intestinal integrity and function, the present study did not directly assess nutrient digestibility, inflammatory status, or feed efficiency. Therefore, the mechanisms underlying the reduced ADG observed in calves with frequent diarrhea remain speculative. Further studies incorporating direct measures of intestinal function and nutrient utilization are warranted to clarify the relationship between diarrhea, GLP-2 secretion, and growth performance in dairy calves.

In conclusion, calves with a greater number of days with diarrhea exhibited lower ADG despite no differences in DMI and tended to have lower plasma GLP-2 concentrations. These findings suggest that frequent diarrhea is associated with impaired growth efficiency and altered intestinal endocrine status. Blood sampling at only 0 and 4 h after Cr-EDTA administration failed to detect differences in intestinal permeability between calves with frequent diarrhea and those with infrequent diarrhea.

## References

[bib1] Araujo G., Yunta C., Terré M., Mereu A., Ipharraguerre I., Bach A. (2015). Intestinal permeability and incidence of diarrhea in newborn calves. J. Dairy Sci..

[bib2] Bertens C.A., Seymour D.J., Penner G.B. (2024). Validation of an in vivo dual permeability marker technique to characterize regional gastrointestinal tract permeability in mid-lactation Holstein cows during short-term feed restriction. J. Dairy Sci..

[bib3] Burrin D.G., Stoll B., Guan X. (2003). Glucagon-like peptide 2 function in domestic animals. Domest. Anim. Endocrinol..

[bib4] Castro J.J., Morrison S.Y., Hosseinni A., Loor J.J., Drackley J.K., Ipharraguerre I.R. (2016). Secretion of glucagon-like peptide-2 responds to nutrient intake but not glucose provision in milk-fed calves. J. Dairy Sci..

[bib5] Connor E.E., Kahl S., Elsasser T.H., Baldwin R.L., Fayer R., Santin-Duran M., Sample G.L., Evock-Clover C.M. (2013). Glucagon-like peptide 2 therapy reduces negative effects of diarrhea on calf gut. J. Dairy Sci..

[bib6] Deng G., Lei Q., Gao X., Zhang Y., Zheng H., Bi J., Wang X. (2021). Glucagon-like peptide-2 modulates enteric Paneth cells immune response and alleviates gut inflammation during intravenous fluid infusion in mice with a central catheter. Front. Nutr..

[bib7] Eckert E., Brown H.E., Leslie K.E., DeVries T.J., Steele M.A. (2015). Weaning age affects growth, feed intake, gastrointestinal development, and behavior in Holstein calves fed an elevated plane of nutrition during the preweaning stage. J. Dairy Sci..

[bib8] Elsabagh M., Inabu Y., Obitsu T., Sugino T. (2017). Response of plasma glucagon-like peptide-2 to feeding pattern and intraruminal administration of volatile fatty acids in sheep. Domest. Anim. Endocrinol..

[bib9] Ezaki T., Kaminishi K., Tamaoku K. (1991). Various acid pretreatments of water samples containing chromium. Bunseki Kagaku.

[bib10] Heinrichs A.J., Heinrichs B.S. (2011). A prospective study of calf factors affecting first-lactation and lifetime milk production and age of cows when removed from the herd. J. Dairy Sci..

[bib11] Inabu Y., Fischer A., Song Y., Guan L.L., Oba M., Steele M.A., Sugino T. (2018). Short communication: The effect of delayed colostrum feeding on plasma concentrations of glucagon-like peptide 1 and 2 in newborn calves. J. Dairy Sci..

[bib12] Khan M.A., Bach A., Weary D.M., von Keyserlingk M.A.G. (2016). Invited review: Transitioning from milk to solid feed in dairy heifers. J. Dairy Sci..

[bib13] Maruta K., Takajo T., Akiba Y., Said H., Irie E., Kato I., Kuwahara A., Kaunitz J.D. (2020). GLP-2 acutely prevents endotoxin-related increased intestinal paracellular permeability in rats. Dig. Dis. Sci..

[bib14] Pisoni L., Devant M., Blanch M., Pastor J.J., Marti S. (2021). Optimization of intestinal permeability assays to study the degree of fasting in gut permeability of unweaned Angus-Holstein bull calves. J. Anim. Sci..

[bib15] Renaud D.L., Buss L., Wilms J.N., Steele M.A. (2020). Technical note: Is fecal consistency scoring an accurate measure of fecal dry matter in dairy calves?. J. Dairy Sci..

[bib16] Smith G.W., Berchtold J. (2014). Fluid therapy in calves. Vet. Clin. North Am. Food Anim. Pract..

[bib17] Soberon F., Van Amburgh M.E. (2013). Lactation Biology Symposium: The effect of nutrient intake from milk or milk replacer of preweaned dairy calves on lactation milk yield as adults: A meta-analysis of current data. J. Anim. Sci..

[bib18] Taylor-Edwards C.C., Burrin D.G., Matthews J.C., McLeod K.R., Holst J.J., Harmon D.L. (2010). Expression of mRNA for proglucagon and glucagon-like peptide-2 (GLP-2) receptor in the ruminant gastrointestinal tract and the influence of energy intake. Domest. Anim. Endocrinol..

[bib19] USDA-APHIS-VS-NAHMS. 2014. Health and management practices on U.S. dairy operations, 2014: Part III—Health and health management on U.S. dairy operations. USDA-APHIS-VS-NAHMS, Fort Collins, CO.

[bib20] Walker M.P., Evock-Clover C.M., Elsasser T.H., Connor E.E. (2015). Short communication: Glucagon-like peptide-2 and coccidiosis alter tight junction gene expression in the gastrointestinal tract of dairy calves. J. Dairy Sci..

[bib21] Wilms J.N., Ghaffari M.H., Darani P.S., Jansen M., Sauerwein H., Steele M.A., Martín-Tereso J., Leal L.N. (2024). Postprandial metabolism and gut permeability in calves fed milk replacer with different macronutrient profiles or a whole milk powder. J. Dairy Sci..

[bib22] Zakia L.S., Gomez D.E., Steele M.A., Constable P.D., LeBlanc S.J., Renaud D.L. (2025). Investigating gut permeability in neonatal calves with diarrhea: A case-control study. JDS Commun..

